# Rasterstereography a reliable tool in scoliosis measurement – a critical review of literature

**DOI:** 10.1186/1748-7161-9-S1-O5

**Published:** 2014-12-04

**Authors:** Narasimman Swaminathan, Arun Mathew Cyriac, Michelle E Lobo

**Affiliations:** 1 Father Muller Medical College, Mangalore, India

## 

Measuring the structural deformities of the spine is always a challenge for the clinician, since it is important to make clinical decision. When repeated exposure to the X ray has to be reduced in the routine practice, rehabilitation professionals are looking for a tool to be used in daily practice. Rasterstereography is one such method used from the early of 1980s attracted the attention of clinician in the western world. Recently it has been introduced to the developing countries like India. In this context it is important to analyse the available evidence pertaining to rasterstereography.

## Aim

The aim of this paper is to review the available published papers and critically analyse the results in terms of reliability, validity and clinical use of this tool.

## Method

A structured literature review was done in PubMed, Ovid, Embase and CINHAL data base using rasterstereography, scoliosis and spine measurement as key words. Boolean logic was used to combine and restrict the results. The articles were restricted to English language. All the articles were appraised by the authors independently by using a predetermined review sheet, which consisted of details including population studied, number subjects, study method, statistical analysis used, results and critical view of the authors.

## Results

20 articles were identified after the search, of which 7 studies used rasterstereography in scoliosis population. There are studies which compared the rasterstereography with gold standard radio graphic measurement. Results of the high quality studies explored the utility of this tool in the evaluation of the scoliosis. This paper will discuss the advantages and challenges of using this tool in daily clinical practice.

## Conclusion

This critical review identifies the advantages of rasterstereography in scoliosis assessment and the future direction in using this novel too in the diagnosis and the management of scoliosis.

